# Teaching Bioinformatics at the Secondary School Level

**DOI:** 10.1371/journal.pcbi.1002242

**Published:** 2011-10-27

**Authors:** Fran Lewitter, Philip E. Bourne

**Affiliations:** 1 Bioinformatics and Research Computing, Whitehead Institute, Cambridge, Massachusetts, United States of America; 2 Skaggs School of Pharmacy and Pharmaceutical Science, University of California San Diego, La Jolla, California, United States of America

Bioinformatics is now an integral part of biology and biological research. The field began with a few people from other disciplines teaching themselves and each other the techniques that are now considered commonplace. These pioneers then began graduate programs [1]–[3] to educate the next generation. Those early graduate students typically came as bench biologists or as computer scientists, both groups requiring significant time to “hybridize”. Not surprisingly, this then led to undergraduate majors in bioinformatics to better prepare students for graduate school and research careers in bioinformatics. In addition, teaching bioinformatics in undergraduate biology classes is also a priority [4], [5]. Through the Education section of *PLoS Computational Biology* we have tried to support this evolution through a collection of educational articles pertinent to the undergraduate level and beyond. It is only natural that we would take the next step [6].

We now introduce a subsection of the Education section with articles devoted to teaching bioinformatics in secondary schools that is derived from the work of the Education committee of the International Society for Computational Biology (ISCB), who identified a need to address the issue of incorporating bioinformatics into secondary school biology classes. They also recognized the interest among researchers to build and participate in outreach programs at the secondary school level given that many funding agencies worldwide encourage such a component in grant applications.

To move the ball forward on secondary school bioinformatics education, at ISCB's 2010 international conference, Intelligent Systems in Molecular Biology (ISMB), the ISCB Education committee organized a half-day tutorial aimed at secondary school biology and chemistry teachers in the Boston area interested in learning about bioinformatics and how to include it in their curricula. The tutorial also attracted researchers involved in organizing or formulating outreach programs in their community. The main focus of the ISMB tutorial was the presentation of lesson plans by a secondary school teacher (David Form, a biology teacher at Nashoba Regional High School, Bolton, Massachusetts) who has successfully incorporated bioinformatics into his courses for more than five years. His is one example of such an effort and is embraced in the Ten Simple Rules and its supplementary material found in this issue. Also in this issue we have an article by Suzanne Gallagher and colleagues on the experience of teaching secondary school level bioinformatics in Boulder, Colorado.

There are many examples of outreach efforts to high school students that we would like to feature in coming months, which incorporate bioinformatics into their programs (see Table 1).

**Table 1:** Examples of Online Resources and Outreach Programs.

**Center for Computational Research at SUNY Buffalo**	http://www.ccr.buffalo.edu/display/WEB/Outreach
**Cold Spring Harbor Laboratory Dolan Learning Center**	http://www.dnalc.org/programs/fieldtrips/hsbioinform.html
**CusMiBio, University of Milan, Italy ** [**9**]	http://www.cusmibio.unimi.it/english03.html
**Harvard University Life Sciences/HHMI (see Microbiology**→**Lesson Plans**→**Recreating the Tree of Life Using Bioinformatics)**	http://outreach.mcb.harvard.edu/materials.htm
**International Society for Computational Biology**	http://www.iscb.org/high-schoolsecondary-school-resources
**Netherlands Bioinformatics Centre**	http://www.bioinformaticsatschool.eu/
**Northwest Association for Biomedical Research**	http://www.nwabr.org/education/itest.html
**University of British Columbia: The Educational Facilities of the Michael Smith Labs**	http://www.bioteach.ubc.ca/tag/bioinformatics/ http://www.bioteach.ubc.ca/genetics-fieldtrips/
**Washington University Saint Louis Science Outreach**	http://www.so.wustl.edu/
**Whitehead Institute Bioinformatics Education Page**	http://jura.wi.mit.edu/bio/education/

There are many other examples of educators doing similar work in school districts worldwide. A recent issue of *Briefings in Bioinformatics* was dedicated to bioinformatics education [7] with a specific example of programs for secondary school students [8], [9].The ISCB Education committee is building a resource of information useful to secondary school teachers who would like to incorporate bioinformatics into their curriculum. In addition, the committee has begun to explore how to include bioinformatics in Advanced Placement courses and exams in the United States, which we also hope to feature in the Education section of the journal.

We encourage feedback of any form, including comments on this editorial, and hearing about your experience teaching bioinformatics to secondary school students.
